# Predicting human papillomavirus vaccine uptake in men who have sex with men the influence of vaccine price and receiving an HPV diagnosis

**DOI:** 10.1186/s12889-021-12396-y

**Published:** 2022-01-06

**Authors:** Po-Yi Yao, Chung-Ying Lin, Nai-Ying Ko, Huachun Zou, Chia-Wen Lee, Carol Strong

**Affiliations:** 1 Department of Public Health, College of Medicine, National Cheng Kung University, Tainan, Taiwan; 2 Department of Occupational Therapy, College of Medicine, National Cheng Kung University, Tainan, Taiwan; 3 Institute of Allied Health Sciences, College of Medicine, National Cheng Kung University, Tainan, Taiwan; 4 Department of Nursing, College of Medicine, National Cheng Kung University, Tainan, Taiwan; 5 School of Public Health (Shenzhen), Sun Yat-Sen University, Shenzhen, China; 6 Kirby Institute, University of New South Wales, Sydney, Australia; 7 Fengshan Lee Chia Wen Urologic Clinic, Kaohsiung, Taiwan

**Keywords:** Men who have sex with men, Papillomavirus vaccine, Intention, HPV screening, Vaccine uptake

## Abstract

**Background:**

To understand how human papillomavirus (HPV) screening results, HPV-related knowledge and attitudes are related to vaccination intention in three cost ranges and the actual vaccination behavior in a community sample of men who have sex with men (MSM).

**Methods:**

MSM aged 20 years of age or older were recruited between October 2015 and May 2016 from community health centers that provide HIV testing and consultation services in Southern Taiwan and on social media. MSM were seen at baseline and again at 6 months after baseline in a cohort study. The baseline study included 253 individuals; 182 of them returned for the 6th-month follow-up. At each visit, MSM were asked to receive HPV screening and filled out a questionnaire. Structural equation modeling was used to test whether attitudinal factors and HPV screening results from the baseline affect their self-reported actual vaccine uptake at the in 6^th^-month follow-up.

**Results:**

Our research included 171 participants from the cohort because they had full information of the study variables (mean ± SD age = 29.21 ± 6.18). Our model showed good model fit using indices such as the comparative fit index (value = 0.998) and root mean square error of approximation (value = 0.013). HPV knowledge can predict those who have intention to take up HPV vaccine no matter what the price (*p* = .02), and then predict vaccine uptake at the follow-up (*p* < .001). A positive HPV screening result can predict vaccine uptake at the follow-up (*p* = .004).

**Conclusion:**

Our findings highlight the impact of vaccine price and HPV screening results on the intention and uptake of HPV vaccine. It is important to raise awareness of HPV in male populations. Clinicians and health educators should establish a safe and private environment for male patients for inquiring about HPV vaccine and HPV-related cancers.

**Supplementary Information:**

The online version contains supplementary material available at 10.1186/s12889-021-12396-y.

## Background

HPV vaccines can effectively protect men from penile cancer, genital warts, and anal and oropharyngeal cancers for both men and women [31]. Current global recommendations for HPV vaccination are mostly for women, yet some countries such as Australia have started to provide a nationwide, free vaccination program for boys [5]. In the U.S., it is recommended that adolescents aged 11–12 receive HPV vaccine, but also both men and women can be vaccinated as early as age 9 through age 26 [4]. Adults aged 27–45 may get the HPV vaccine if they and their clinicians find it suitable [4]. In Taiwan, since 2019, free vaccines have been provided only for girls aged 13. In Taiwan it is recommended that women ages 9–45 and men ages 9–26 receive HPV vaccine [18]. Discussions related to providing HPV vaccine for men who have sex with men (MSM) are increasing [14, 29, 33]. Nevertheless, the current recommendation of vaccination programs in most countries focuses only on girls,thus, MSM are the least protected population regarding the benefits of HPV vaccine.

A policy to encourage providing HPV vaccine for MSM faces some challenges. One is the concern regarding cost effectiveness, although studies in England and Australia have indicated the benefits of HPV vaccinations in MSM [22, 45]. Another concern is the lack of awareness regarding HPV and HPV vaccine in men (including MSM), and even less awareness in the Asia–Pacific region. Very few studies have been published in Asia regarding HPV knowledge and risk perceptions among MSM. Researchers in Hong Kong surveyed 542 MSM with wide age variation (range 18–60 years) and found that a large proportion had never heard of HPV (46.7%). Common misconceptions in this population included: (1) they do not know that HPV affects men, and (2) they think that HPV can be controlled by antibiotics [19]. Countries’ and regions’ lack of data on HPV knowledge and risk perceptions in MSM populations reflects an oversight of the importance of HPV vaccination in MSM populations, and can continue affecting the vaccine policy in the long term. Thus, to facilitate appropriate policy implementation, more research is needed to assess the current status for vaccine awareness and the factors associated with vaccine uptake.

We used the Health Belief Model (HBM) and the Theory of Planned Behavior (TPB) in understanding the uptake of HPV vaccine in men [13, 14, 27]. Factors based on cognitive constructs in these two theories were cited in the following studies: perceived HPV vaccine benefits [13, 14, 28, 41, 44], subjective norms [13, 14, 28, 40, 44], perceived severity [19, 20, 28, 40, 44], and perceived barriers [13, 19, 28, 41, 44]. Studies have combined these two theories and extracted constructs to predict the vaccine uptake in men [13, 14]. Studies using HBM and TPB found that the perceived severity of HPV disease and the perceived barriers to HPV vaccine can predict the acceptability of HPV vaccines in MSM [19],the perceived benefits and cost of vaccine, and perceived norms for vaccination can predict HPV vaccine acceptability for young men [13],two items of perceived susceptibility and one item of perceived severity are associated with the intention to take up HPV vaccine in the next six months for MSM in Hong Kong [40]. To date, HBM and TPB have been the most frequently used theories to predict HPV vaccine uptake in men or MSM in the literature and have been generally found effective due to the above-mentioned constructs that were significantly associated with vaccine uptake.

Despite the factors derived from risk perceptions and attitudes toward the HPV vaccine having been tested in several studies [13, 14, 28], two factors—vaccine cost and HPV screening results—were rarely included in understanding the decision-making process in HPV vaccine uptake in MSM. To our knowledge, the only study that employed actual HPV screening results in understanding HPV vaccine uptake in MSM found that self-reported HPV test results elicited negative emotional responses and then increased the intent to get vaccinated [42]. Yet that study only assessed the intention for HPV vaccine but not the actual vaccine behavior. Partly due to the higher cost, HPV screenings are not included in routine sexually transmitted infection (STI) checkups. Even the most thorough STI panel testing—which includes chlamydia, gonorrhea, hepatitis, herpes, HIV, and syphilis—does not contain HPV tests, despite the finding that anal HPV infection prevalence can go as high as 69%–77% in MSM [10, 36, 36].

HPV vaccine uptake as a type of self-care intervention for sexual health is also influenced by the HPV vaccine prices. Countries with higher income have a higher rate of introducing HPV vaccine with wider coverage [2]. The affordability of the HPV vaccine may likely be one major reason for MSM to have the intention or actually receiving HPV vaccine [34]. The present study’s aim was to understand how HPV screening results, HPV-related knowledge, and HPV vaccine attitudes are related to the intention to receive the vaccine and the actual vaccination behavior in a community sample of MSM. We further tested the MSM’s acceptance regarding the different costs of HPV vaccine by asking their willingness to receive HPV vaccine in three cost ranges.

## Methods

### Participants and study design

Participants were recruited between October 2015 and May 2016 from community health centers that provide HIV testing and consultation services in Kaohsiung City in Southern Taiwan and on social media. Eligible participants were 20 years of age or older, had ever had sex (including mutual masturbation, oral sex, or anal sex) with another man, and were willing to give written consent to participate in the cohort study. There were no exclusion criteria. We included participants who missed HPV vaccines at the appropriate recommended ages because they can still be exposed to new HPV infection. If the MSM’ clinicians had suggested that they receive HPV vaccine, it was still possible for them to receive the vaccine even though it may not be as beneficial as receiving the vaccine at a younger age. MSM were seen at baseline and again at 6 months. We used baseline and 6^th^-month data for this study. The baseline study included 253 individuals; 182 of them returned for the 6^th^-month follow-up. In both visits, participants were asked to receive HPV screening and filled out a questionnaire. Survey questions included assessments of HPV knowledge, HPV vaccine attitudes, and intention to take up HPV vaccine at different prices. Penile and anal HPV screening was used to detect HPV infection involving 37 genotypes (Roche Molecular Diagnostics, Pleasanton, California, USA). Screening results were emailed to each participant within a month after the baseline, along with an information sheet that explained how to interpret the results; HPV vaccine as a preventive measure was briefly mentioned. Only the screening results from the baseline were used for our study. At the 6^th^-month follow-up, participants were asked whether they had actually received HPV vaccine. The study was approved by the Ethics Committee of the National Cheng Kung University Hospital (reference number: A-BR-103–075). More details regarding the cohort description and baseline findings are reported elsewhere [38, 39]. In this present paper, we analyzed 171 participants from the cohort because they had full information of the study variables.

### Instruments

Demographics included age, relationship status, education, occupation and monthly income.

HPV knowledge was based on 28 items from the original scale that included questions such as “having many sexual partners increases the risk of HPV infection” and “HPV vaccines are most effective if given to people who've never had sex” developed by Perez et al. to measure HPV knowledge (Cronbach's α: 0.739-0.916) [30]. Participants received one point for each correct answer. “Don’t know” responses or missing responses were coded as incorrect. A higher total score represented a higher level of HPV knowledge.

HPV-related and HPV vaccine attitude included five constructs: capacity to obtain HPV-related information, perceived severity of HPV disease, subjective norm, perceived benefits of HPV vaccine, and perceived barriers to HPV vaccine. All questions were asked on a five-point scale from 1 “stronger disagree” to 5 “stronger agree.” The mean score represented the level of each construct of HPV vaccine attitude. Details regarding each construct are listed below.

Capacity to obtain HPV-related information used three questions that included whether the participants had enough information about HPV vaccine, whether they thought there was enough research about the HPV vaccine, and whether they were comfortable discussing sexual health issues or HPV vaccines with healthcare providers (Cronbach's α in the present study = 0.780; Cronbach's α = 0.64-0.84 in previous studies) [9, 19, 35].

Perceived severity of HPV disease was based on three questions that included whether the participants felt that contracting HPV, genital warts, or HPV-induced cancer was a serious problem (Cronbach's α in the present study = 0.893; Cronbach's α = 0.78 in previous studies) [19, 23, 23].

Subjective norm was based on four questions that included whether they believed in the advice from healthcare providers and government regarding HPV vaccine, and whether they felt that recommendations for HPV vaccination from healthcare providers and government were important (Cronbach's α in the present study = 0.877) (Bowyer, Forster, Marlow, & Waller, 2014).

Perceived benefits of HPV vaccine were based on five questions that included whether participants thought that the HPV vaccine could prevent HPV, genital warts, or HPV-induced cancer, whether they thought that the HPV vaccine could protect their sexual health, and whether they thought that the HPV vaccine could protect their partners from every type of HPV (Cronbach's α in the present study = 0.865; Cronbach's α = 0.57-0.84 in previous studies) [19, 23, 30, 35].

Perceived barriers included eight questions such as concerns regarding the vaccine’s safety and whether the participants thought that it was hard to find a provider or clinic to receive the HPV vaccine (Cronbach's α in the present study = 0.786; Cronbach's α = 0.64-0.84 in previous studies) [1, 9, 12, 35].

Intention to receive the HPV vaccine was assessed by one question: whether the participants were willing to take up HPV vaccine within six months given the efficacies in preventing genital warts, penile and anal cancers, conditioned on three price range possibilities: (a) NT$8000 to 12,000 (equals US$ 267 to 400), (b) NT$4000 to 8000 (US$ 133 to 267), or (c) free for a total of 3 shots. This question was revised from previous research but used price ranges that matched the prices in Taiwan [19]. For each statement, participants responded on a five-point scale (from 1 “stronger disagree” to 5 “stronger agree”). We categorized all participants into three groups: those who had intention to take up HPV vaccine no matter what the price (up to NT$12,000),those who had intention to take up HPV vaccine if the price was below NT$8000; those who had no intention even if it was provided free, or those who had the intention only when the vaccine was free. Detailed questions are provided in Supplementary file 1.

Vaccine uptake was assessed in the 6^th^ month follow-up. We asked participants whether they had received any HPV shot in the past six months. As long as they had initiated the first shot, they were considered as having received HPV vaccine in the follow-up.

### Data analysis

We used mean and percentage to present the descriptive statistics for participant characteristics, HPV knowledge, HPV vaccine attitude, HPV infection, intention and uptake. Then we used independent *t-*test and *χ*^*2*^ tests to compare the characteristics, HPV knowledge, HPV vaccine attitude, HPV infection, and intention between those who reported having received the HPV vaccine at the 6^th^-month follow-up and those who had not. We used Pearson’s correlation to compute the constructs between HPV vaccine attitude, HPV infection, intention, and uptake.

**Fig. 1:**
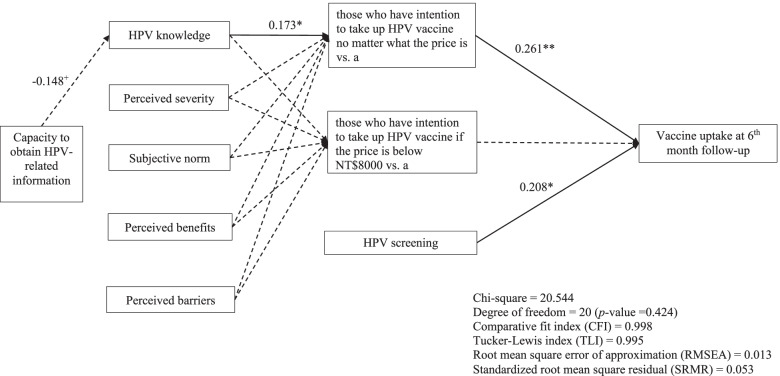
Standardized solutions for the structural model of vaccine attitude and knowledge, intention with different vaccine price range, HPV screening, and HPV vaccine uptake

Our research established the model that HPV knowledge and vaccine attitude can predict intention to take up vaccine with a different price and that HPV screening results and intention can predict HPV vaccine uptake. Structural equation modeling (SEM) was applied to test the model about HPV vaccine uptake and factors associated with intention among MSM (Fig. 1). Comparative fit index (CFI), Tucker-Lewis Index (TLI), root mean square error of approximation (RMSEA), and standardized root mean square residual (SRMR) were adopted to determine the data-model fit: CFI and TLI > 0.9 together with RMSEA and SRMR < 0.08 indicate satisfactory fit [17]. The descriptive statistics and the correlation were determined using SPSS 20.0,SEM was performed using AMOS.

## Results

### Participant characteristics of MSM and descriptive results

Table 1 presents participant characteristics at baseline stratified by HPV vaccine uptake in the 6^th^-month follow-up. Out of 171 MSM, 34.5% had anal or penile HPV DNA positivity, 62% were below age 30, 76% had a college degree or below. Almost 60% were single or single but dating; the rest were in committed relationships. About two thirds were employed. Twenty-eight had received HPV vaccine at the 6^th^-month follow-up (16.4%). As for intention, 45% were willing to take up HPV if the price was NT$8000 to 12,000, 60% if the price was NT$4000 to 8000, and 93% if the vaccine was free.

**Table 1:** Participant characteristics at baseline (*N* = 171)

	**All**	**HPV vaccine uptake in the 6**^**th**^** month follow-up**		**Different between vaccine uptake or not**
		**Yes** (16.4%)	**No** (83.6%)	
	n (%) or mean ± SD	n (%) or mean ± SD		*p*-value
**Age(years)**	29.21 ± 6.18	29.71 ± 6.72	29.12 ± 6.1	0.641
< 20	1 (0.6)	0(0)	1(0.7)	
20–30	105 (61.4)	16(57.1)	89(62.2)	
30–40	51(29.8)	9(32.1)	42(29.4)	
> 40	14 (8.2)	3(10.7)	11(7.7)	
**Relationship status**				0.834
Single or single but dating	99(58.2)	17(60.7)	82(57.8)	
In a committed relationship	71(41.8)	11(39.3)	60(42.2)	
**Education**				0.092
College/High school	130(76)	18(64.3)	112(78.3)	
Graduate school	41(24.0)	10(35.7)	31(21.7)	
**Occupation**				0.478
Employed	112(65.5)	19(67.9)	93(65.0)	
Student/Unemployed	59(34.5)	9(32.1)	50(35)	
**Average income over the past year**				0.059
≤ NT$19,999	56(32.8)	9(32.1)	47(32.9)	
NT$20,000–39,999	71(41.5)	5(17.9)	66(46.2)	
≥ NT$40,000	44(25.7)	14(50.0)	30(21.0)	
**HPV knowledge**	17.48 ± 5.85 (62.4)	19.43 ± 5.41 (69.4)	17.1 ± 5.87 (61.1)	0.054
**Capacity to obtain HPV-related information**	3.14 ± 0.91	2.89 ± 0.99	3.18 ± 0.89	0.123
**Perceived severity of HPV**	4.15 ± 0.83	4.14 ± 0.78	4.16 ± 0.84	0.938
**Subjective norm**	4.07 ± 0.75	4.15 ± 0.52	4.05 ± 0.78	0.529
**Perceived benefits of HPV vaccine**	3.90 ± 0.69	3.99 ± 0.61	3.88 ± 0.71	0.466
**Perceived barriers of HPV vaccine**	2.53 ± 0.55	2.42 ± 0.60	2.55 ± 0.54	0.287
**HPV positive**	59(34.5)	16(57.1)	43(30.1)	0.006**
**Intention to receive the HPV vaccine with three price range**				
NT8000-12,000	77(45.0)	21(75.0)	56(39.2)	< 0.001***
NT4000-8000	103(60.2)	23(82.1)	80(55.9)	0.007**
free	159(93.0)	26(93.0)	133(93.0)	0.667
**Intention to receive the HPV vaccine**				0.003**
No matter what the price is	77(45.0)	21(75.0)	56(39.2)	
If the price is below NT$8000	26(15.2)	2(7.1)	24(16.8)	
If the price free or no intention	68(39.7)	5(17.8)	63(44.1)	

*p < 0.05, ** *p* < 0.01, *** *p* < 0.001

To summarize, 45% of the MSM had intention to take up HPV vaccine no matter what the price; 15.2% had intention to take up HPV vaccine if the price was below NT$8000; 39.7% of the MSM had no intention even if it were provided free or had intention only when the vaccine was free. Comparing MSM who reported having HPV vaccine at the 6^th^-month follow-up with those who did not, only HPV positive and intention to receive the HPV vaccine were significantly associated (Table 1). Three quarters of the MSM who had initiated HPV vaccine in the follow-up were those with high intention at baseline to receive HPV no matter what the price.

In Table 2, we present the questions of HPV knowledge, such as transmission route, prevention methods, treatment and related consequences. More than 90% of the MSM knew that men could get HPV (92.4%), that having many sexual partners increased the risk of getting HPV (93.6%), and that HPV could be transmitted through anal sex (92.4%). The worst correct rates of HPV knowledge were that HPV cannot cause herpes (7%), HPV usually does not need any treatment (8.8%), and HPV cannot cause rectal cancer (14.6%). The overall correct rate of HPV knowledge was 62.4% (Table 1). Table 3 presents the correlations among the variables used in our SEM model. HPV knowledge was correlated positively with perceived benefits of HPV vaccine (r = 0.198), and negatively with capacity to obtain HPV-related information (r = -0.214), and perceived barriers of HPV vaccine (r =

**Table 2:** HPV knowledge: answering correctly (*n* = 171)

Item	**Correct answer** n(%)
HPV is very rare. (F)	119 (69.6)
HPV always has visible signs or symptoms. (F)	75(43.9)
HPV can be transmitted through genital skin-to-skin contact. (T)	139(87.1)
There are many types of HPV. (T)	126(73.7)
HPV can cause HIV/AIDS. (F)	105(61.4)
HPV can cause genital warts. (T)	151(88.3)
Men cannot get HPV. (F)	158(92.4)
Using condoms reduces the chances of HPV transmission. (T)	145(84.8)
HPV can be cured with antibiotics. (F)	61(35.7)
Having many sexual partners increases the risk of getting HPV. (T)	160(93.6)
HPV usually doesn't need any treatment. (T)	15(8.8)
Most sexually active people will get HPV at some point in their lives. (T)	124(72.5)
Having sex at an early age increases the risk of getting HPV. (T)	75(43.9)
HPV can cause cancer of the penis or anal. (T)	123(71.9)
HPV can cause cancer of rectum. (F)	25(14.6)
HPV is a bacterial infection. (F)	90(52.6)
HPV can be transmitted through oral sex. (T)	150(87.7)
HPV can cause herpes. (F)	12(7.0)
HPV can be transmitted through anal sex. (T)	158(92.4)
HPV infections always lead to health problems. (F)	45(26.3)
A person with no symptoms cannot transmit the HPV infection. (F)	136(79.5)
HPV can cause cervical cancer. (T)	133(77.8)
A person could have HPV for many years without knowing it. (T)	141(82.5)
The HPV vaccines offer protection against all sexually transmitted infections. (F)	97(56.7)
The HPV vaccines are most effective if given to people who've never had sex. (T)	80(46.8)
One of the HPV vaccines offers protection against genital warts. (T)	139(81.3)
The HPV vaccine protects you from every type of HPV. (F)	97(56.7)
You can cure HPV by getting the HPV vaccine. (F)	100(58.5)
Correct answer of each items (T):Ture; (F):False

**Table 3:** Correlation matrix among HPV knowledge, vaccine attitude, screening, intention, and vaccine uptake (*n* = 171)

		1	2	3	4	5	6
1	**HPV knowledge**	—					
2	**Capacity to obtain HPV-related information**	-0.213^**^	—				
3	**Perceived severity of HPV**	-0.008	0.061	—			
4	**Subjective norm**	0.057	-0.008	0.405^**^	—		
5	**Perceived benefits of HPV vaccine**	0.198^**^	-0.007	0.268^**^	0.534^**^	—	
6	**Perceived barriers of HPV vaccine**	-0.301^**^	0.431^**^	-0.119	-0.335^**^	-0.356^**^	—
*p < 0.05, ** *p* < 0.01, *** *p* < 0.001							

-0.301). Capacity to obtain HPV-related information was correlated positively with perceived barriers to HPV vaccine (r = 0.431). Perceived severity of HPV was correlated positively with subjective norm (r = 0.405) and perceived benefits of HPV vaccine (r = 0.268). Subjective norm was correlated positively with perceived benefits of HPV vaccine (r = 0.534). Perceived benefits of HPV vaccine were negatively correlated with perceived barriers to HPV vaccine (r = -0.356). We provided results of a regression analysis to determine what percent of the variance of the outcome variable (vaccine uptake) was determined by the different independent variables in Supplementary file 2.

### Structural model

Our model as shown had good fit to the data (CFI = 0.998; TLI = 0.995; RMSEA = 0.013; SRMR = 0.053). The research use of type I error at 0.05, degree of freedom at 20, sample size at 171, null RMSEA at 0.00, and alternative RMSEA at 0.08, the power of the model was 0.82. The value of *R*^2^ are the following: 0.022 for HPV knowledge, 0.064 for those who have intention to take up HPV vaccine no matter what the price is versus those who have no intention even if it were provided free or those who only have intention when the vaccine is free, 0.022 for those who have intention to take up HPV vaccine if the price is below NT$8000 versus those who have no intention even if it were provided free or those who only have intention when the vaccine is free, and 0.113 for HPV vaccine uptake.

Three paths were significant: (1) HPV knowledge can predict those who have intention to take up HPV vaccine no matter what the price (standardized coefficient = 0.173, *p* < 0.05). (2) Those who have intention to take up HPV vaccine no matter what the price can predict vaccine uptake at the 6^th^-month follow-up (standardized coefficient = 0.261, *p* < 0.001). (3) HPV screening can predict vaccine uptake at the 6^th^-month follow-up (standardized coefficient = 0.208, *p* < 0.05). One path was marginally significant: from capacity to obtain HPV-related information to HPV knowledge (standardized coefficient = -0.148, *p* < 0.1). There were no significant differences between perceived severity of HPV disease, subjective norm, perceived benefits, and barriers to intention to receive the vaccine in the 6^th^-month follow-up (Table 4). The direct**,** indirect, and total effects of the variables are in Supplementary file 3.

**Table 4:** Standardized path coefficients for the structural model

Independent variable/Mediator	Mediator/Dependent variable	β	S.E	t-value	*p*-value
Capacity to obtain HPV-related information	HPV knowledge	-0.148	0.485	-1.953	0.051
HPV knowledge	Those who have intention to take up HPV vaccine no matter what the price is vs. the reference^a^	0.173	0.006	2.329	0.020
Perceived severity	Those who have intention to take up HPV vaccine no matter what the price is vs. the reference^a^	0.016	0.054	0.174	0.862
Subjective norm	Those who have intention to take up HPV vaccine no matter what the price is vs. the reference^a^	0.079	0.067	0.791	0.429
Perceived benefits	Those who have intention to take up HPV vaccine no matter what the price is vs. the reference^a^	0.020	0.066	0.220	0.826
Perceived barriers	Those who have intention to take up HPV vaccine no matter what the price is vs. the reference^a^	-0.119	0.070	-1.536	0.125
HPV knowledge	Those who have intention to take up HPV vaccine if the price is below NT$8000 vs. the reference^a^	0.059	0.005	0.770	0.441
Perceived severity	Those who have intention to take up HPV vaccine if the price is below NT$8000 vs. the reference^a^	0.059	0.040	0.389	0.697
Subjective norm	Those who have intention to take up HPV vaccine if the price is below NT$8000 vs. the reference^a^	0.112	0.049	0.751	0.453
Perceived benefits	Those who have intention to take up HPV vaccine if the price is below NT$8000 vs. the reference^a^	0.043	0.060	1.090	0.276
Perceived barriers	Those who have intention to take up HPV vaccine if the price is below NT$8000 vs. the reference^a^	0.066	0.052	0.832	0.405
Those who have intention to take up HPV vaccine no matter what the price is vs. the reference^a^	Vaccine uptake in the 6^th^ month follow-up	0.261	0.059	3.323	< 0.001
Those who have intention to take up HPV vaccine if the price is below NT$8000 vs. the reference^a^	Vaccine uptake in the 6^th^ month follow-up	-0.006	0.081	-0.080	0.936
HPV screening	Vaccine uptake in the 6^th^ month follow-up	0.208	0.056	2.884	0.004

β is the standardized path coefficient, *S.E.* is the standard error and *p* is the significance level

^a^Reference group: those who have no intention even if it was provided free or those who only have intention when the vaccine is free

## Discussion

To the best of our knowledge, this is the first study to explore HPV vaccine uptake and associated factors such as HPV knowledge, vaccine attitude, and price among MSM in Taiwan. Two factors were directly linked to whether MSM actually initiated HPV vaccine in the following six months: a positive HPV screening result and those with high intention to receive vaccine regardless of the price. HPV knowledge was only associated with vaccine uptake by individuals with high intention to receive vaccine regardless of the price, but not with those with lower intention, determined by the willingness of receiving vaccine only at a lower price. Improving HPV knowledge and lowering the vaccine price can be two future directions to improve vaccine uptake among MSM.

Consistent with previous studies, knowing one’s own STI status can affect HPV vaccine uptake, including non-HPV STIs such as chlamydia, gonorrhea, syphilis, or HIV (Meites, Markowitz, Paz-Bailey, & Oster, 2014), and self-reported HPV screening results or genital warts [7, 26, 42]. Studies have shown a low awareness of contracting HPV, specifically among MSM [19]. Given the high price of HPV screening and the low awareness of HPV in men in Taiwan, it is unlikely that participants knew their infection status before our study. HPV, as a type of STI with a long history of “feminization”—in which HPV was over-identified as a disease that specifically matters to women and was usually framed as not important for men [8]—may have come as a shock when MSM learned the results of their HPV infection. HPV diagnosis can be viewed as a threat that prompts individuals to seek out more information for the treatment and prevention of future infections [42]. A policy or recommendation to increase HPV screening or self-tests in men may increase the awareness and vaccine uptake rate in men, and in turn improve the herd immunity that benefits both men and women. Self-collected rectal swabs—although available and with potential to be a sustainable option for HPV control—are less likely to collect enough specimen for cytological interpretation [15]. More studies are needed to evaluate whether providing HPV screening to men is cost effective considering the disease burden and income levels in each country.

The price of HPV vaccine affects an individual’s intention for vaccine uptake [19]. Compared to previous findings in the Asia–Pacific region, our results showed a higher level of intention to receive free vaccination: 93% in our study vs. 80% in Hong Kong in 2013 [19]. A possible reason is that our participants perceived the serious severity of HPV-related disease. The Taiwan government’s promotion of HPV vaccination in females might also have indirectly raised the awareness and perceived threat of HPV among men. If the vaccine price dropped to half of the current price in Taiwan, the intention for HPV vaccine uptake in MSM could increase from 45 to 60%—even to 93%—if the vaccine were completely free. Our study provides a reference for policymakers to determine the range of subsidized HPV vaccination. HPV knowledge predicted vaccine intention and uptake only in MSM who had the intention to take up HPV vaccine regardless of the price, but not for those who had the intention to take up HPV vaccine only if the price were below NT$8000. While the focus has been put on the importance of HPV knowledge in relation to willingness of HPV vaccine uptake in men [28], our study highlighted that the concerns for vaccine price may exceed the positive effect of HPV knowledge. In our sample, those who indicated that they would only take up less expensive vaccine had lower monthly income than those with intention to take up vaccine regardless of the price.

Interventions designed to improve HPV knowledge to increase vaccine uptake in MSM needs to be supported with policies to subsidize HPV vaccine in this population. The insufficient HPV knowledge may result from participants’ low capacity to obtain HPV-related information, a tendency we observed in our data that was only marginally significant. Only 46% of MSM in our sample felt free to discuss HPV vaccine with other people; even fewer (29%) felt free to talk to healthcare providers about sexual health, which may contribute to MSM’s inability to obtain HPV-related information. Because of the major discrimination against MSM (Fontenot, Fantasia, Vetters, & Zimet, 2016; Christopher W [43] and shaming for STI [32], more education is needed for healthcare providers to reduce the stigma and to encourage more communication between MSM and healthcare providers about sexual health. A friendly healthcare environment may also encourage MSM to actively seek HPV vaccine information and improve the uptake rate.

### Limitations

Our findings should be interpreted with the following limitations in mind. First, study participants were recruited only if they were willing to receive an anal and penile swab for HPV screening. Compared to the general MSM population, our study sample might have higher awareness regarding their personal health. One study indicated that men who participated in an HPV study differed from those who did not in the number of receptive anal sex partners and income [15]. It is possible that in the general MSM population with lower self-care intention, there is a stronger association between perceived risk and vaccine uptake. Second, participants who did not come back for the follow-up might have been those with less alertness or motivation to receive the HPV vaccine. The association between vaccine price and intention for uptake may be even stronger in participants who were lost to follow-up. Third, because HPV vaccine is not covered by universal health insurance in Taiwan, we cannot verify the self-reported uptake with health records. The prevalence of actual vaccine uptake might be over-reported. Fourth, HPV-related and HPV vaccine attitude were only measured once at the baseline, but not again after participants received their HPV screening results. Baseline attitudes were not significantly associated with their later vaccine uptake, which is different from previous research [6] in various jurisdictions using regression models that have shown that attitudes and beliefs are associated with MSM's intentions to receive the HPV vaccine [6, 7, 16, 25]. It is likely that their attitudes changed after receiving their HPV screening results. Our study instead provided a perspective of whether attitudes were associated with uptake behavior without the influence of knowing their status.

## Conclusion

In conclusion, our findings highlight the impact of vaccine prices on the intention and uptake of HPV vaccine. Given the benefit of HPV vaccine in both men and women and the potential benefit of herd immunity [21], additional attention might be needed to develop a gender-neutral policy such as a gender-neutral HPV vaccination and catch-up programs to lower the burden of vaccine price and increase awareness of HPV-related health outcomes among men. Interventions that aim to increase knowledge to improve vaccine uptake should not overlook the influence of vaccine price.

Our results can provide some strategies to practice for the future. First, the Taiwan government can inspect the relevance of the price of HPV vaccine and vaccinations. Since the vaccine has been proven to be effective in preventing some types of HPV infection, if the price becomes reasonable and acceptable, the vaccination rate might be increased to achieve more comprehensive protection. Second, the results of HPV tests will affect the willingness of MSM to receive the vaccination, and positive results may improve their alertness to the disease. This highlights the importance of raising awareness of HPV in male populations. Clinicians and health educators should establish a safe and friendly environment for male patients for inquiring about HPV vaccine and HPV-related cancers.

## Supplementary Information

**Additional file 1.** Questionnaire about intention to receive the HPV vaccine.

**Additional file 2.** Regression analysis to determine what percent of the variance of the outcome variable (vaccine uptake) is determined by the different independent variables.

**Additional file 3.** Standardized direct and indirect effects of knowledge, Those who have intention to take up HPV vaccine if the price is below NT$8000 vs. the reference^a^, Those who have intention to take up HPV vaccine no matter what the price is vs. the reference^a^ and Vaccine uptake in the 6th month follow-up.

## Data Availability

The datasets used and/or analysed during the current study available from the corresponding author on reasonable request.

## Abbreviations

CFI: Comparative fit index
HBM: Health Belief Model
HPV: Human papillomavirus
MSM: Men who have sex with men
RMSEA: Root mean square error of approximation
SEM: Structural equation modeling
SRMR: Standardized root mean square residual
TLI: Tucker Lewis Index
TPB: Theory of Planned Behavior
