# Supplementary material for: Predicting human papillomavirus vaccine uptake in men who have sex with men the influence of vaccine price and receiving an HPV diagnosis
Source: BMC Public Health. 2022 Jan 6;22:28. doi: 10.1186/s12889-021-12396-y (PMC8740414; doi:10.1186/s12889-021-12396-y)
Supplement: Supplementary file 1 — Additional file 1. Questionnaire about intention to receive the HPV vaccine. [file 12889_2021_12396_MOESM1_ESM.docx]

**Questionnaire about intention to receive the HPV vaccine**

What do you think of the following description of the intention to receive the HPV vaccine? (Please select the option that best suits you)

| Question | Answer |
| --- | --- |
| If HPV vaccines can effectively prevent from getting genital warts and penile or anal cancer, I have intention to receive three shots in the next six months for a total cost of NTD $ 8000-12000. | strongly disagree(1)/ disagree(2) /  neutral(3)/  agree(4)/  strongly agree(5) |
| If HPV vaccines can effectively prevent from getting genital warts and penile or anal cancer, I have intention to receive three shots in the next six months for a total cost of NTD $ 4000-8000. | strongly disagree(1)/ disagree(2) /  neutral(3)/  agree(4)/  strongly agree(5) |
| If HPV vaccines can effectively prevent from getting genital warts and penile or anal cancer, I have intention to receive three shots in the next six months for free. | strongly disagree(1)/ disagree(2) /  neutral(3)/  agree(4)/  strongly agree(5) |
