# Supplementary material for: Predicting human papillomavirus vaccine uptake in men who have sex with men the influence of vaccine price and receiving an HPV diagnosis
Source: BMC Public Health. 2022 Jan 6;22:28. doi: 10.1186/s12889-021-12396-y (PMC8740414; doi:10.1186/s12889-021-12396-y)
Supplement: Supplementary file 2 — Additional file 2. Regression analysis to determine what percent of the variance of the outcome variable (vaccine uptake) is determined by the different independent variables. [file 12889_2021_12396_MOESM2_ESM.docx]

|  | Vaccine uptake at 6^th^ month follow-up | | | |
| --- | --- | --- | --- | --- |
|  | OR(95%CI) | p | R^2^ | R^2^-change |
| HPV knowledge | 1.042(0.9950-1.143) | 0.386 | 0.024 |  |
| Capacity to obtain HPV-related information | 0.648(0.367-1.144) | 0.134 | 0.033 | 0.009 |
| Perceived severity | 0.930(0.486-1.779) | 0.825 | 0.033 | 0 |
| Subjective norm | 0.925(0.407-2.102) | 0.852 | 0.034 | 0.001 |
| Perceived benefits | 1.271(0.548-2.947) | 0.576 | 0.035 | 0.001 |
| Perceived barriers | 1.396(0.513-3.798) | 0.514 | 0.035 | 0 |
| Those who have intention to take up HPV vaccine no matter what the price is vs. a | 4.829(1.518-15.365) | 0.008 | 0.091 | 0.056 |
| Those who have intention to take up HPV vaccine if the price is below NT$8000 vs. a | 0.911(0.150-5.509) | 0.919 | 0.091 | 0 |
| HPV screening | 3.557(1.439-8.796) | 0.006 | 0.131 | 0.04 |

**Regression analysis to determine what percent of the variance of the outcome variable (vaccine uptake) is determined by the different independent variables**

^a^Reference group: those who have no intention even if it was provided free or those who only have intention when the vaccine is free.
