# Supplementary material for: Predicting human papillomavirus vaccine uptake in men who have sex with men the influence of vaccine price and receiving an HPV diagnosis
Source: BMC Public Health. 2022 Jan 6;22:28. doi: 10.1186/s12889-021-12396-y (PMC8740414; doi:10.1186/s12889-021-12396-y)
Supplement: Supplementary file 3 — Additional file 3. Standardized direct and indirect effects of knowledge, Those who have intention to take up HPV vaccine if the price is below NT$8000 vs. the referencea, Those who have intention to take up HPV vaccine no matter what the price is vs. the referencea and Vaccine uptake in the 6th month follow-up. [file 12889_2021_12396_MOESM3_ESM.docx]

**Standardized direct and indirect effects of knowledge, Those who have intention to take up HPV vaccine if the price is below NT$8000 vs. the reference^a^, Those who have intention to take up HPV vaccine no matter what the price is vs. the reference^a^ and Vaccine uptake in the 6th month follow-up**

| **Variables** | **Knowledge**  **(Total effects=-0.148)** | |  | **Those who have intention to take up HPV vaccine if the price is below NT$8000 vs. the reference^a^**  **(Total effects=0.268)** | |  | **Those who have intention to take up HPV vaccine no matter what the price is vs. the reference^a^**  **(Total effects=0.143)** | |  | **Vaccine uptake in the 6^th^ month follow-up**  **(Total effects=0.499)** | |
| --- | --- | --- | --- | --- | --- | --- | --- | --- | --- | --- | --- |
|  | **Direct**  **effect** | **Indirect**  **effect** |  | **Direct**  **effect** | **Indirect**  **effect** |  | **Direct**  **effects** | **Indirect**  **effect** |  | **Direct**  **effects** | **Indirect**  **effect** |
| HPV screening | - | - |  | - | - |  | - | - |  | 0.208 | 0.000 |
| Capacity to obtain HPV-related information | -0.148 | 0.000 |  | 0.000 | -0.009 |  | 0.000 | -0.026 |  | 0.000 | -0.007 |
| Perceived barriers | - | - |  | 0.066 | 0.000 |  | -0.119 | 0.000 |  | 0.000 | -0.031 |
| Perceived benefits | - | - |  | 0.004 | 0.000 |  | 0.020 | 0.000 |  | 0.000 | 0.005 |
| Subjective norm | - | - |  | 0.112 | 0.000 |  | 0.079 | 0.000 |  | 0.000 | 0.020 |
| Perceived severity | - | - |  | 0.036 | 0.000 |  | 0.016 | 0.000 |  | 0.000 | 0.004 |
| knowledge | - | - |  | 0.059 | 0.000 |  | 0.173 | 0.000 |  | 0.000 | 0.045 |
| Those who have intention to take up HPV vaccine if the price is below NT$8000 vs. the reference^a^ | - | - |  | - | - |  | - | - |  | -0.006 | 0.000 |
| Those who have intention to take up HPV vaccine no matter what the price is vs. the reference^a^ | - | - |  | - | - |  | - | - |  | 0.261 | 0.000 |

^a^Reference group: those who have no intention even if it was provided free or those who only have intention when the vaccine is free.
